# Development and Validation of a Standardized Palpebral Conjunctival Redness Scale

**DOI:** 10.7759/cureus.102092

**Published:** 2026-01-22

**Authors:** Jared W Lim, Calvin W Wong, Nathan A Seto, Harrison L Le, Richard W Yee

**Affiliations:** 1 McGovern Medical School, University of Texas Health Science Center at Houston, Houston, USA; 2 Department of Ophthalmology, University of Texas Health Science Center at Houston, Houston, USA; 3 Ophthalmology, Richard W. Yee, MD PLLC, Bellaire, USA

**Keywords:** bulbar conjunctival redness, conjunctival assessment, grading scale, inter-rater reliability, palpebral conjunctival redness

## Abstract

Purpose: This study aims to develop and validate a standardized palpebral conjunctival redness (PCR) scale for clinical assessment of ocular inflammation, addressing the distinct vascular anatomy and immune characteristics of the palpebral conjunctiva compared to the bulbar conjunctiva.

Methods: A study was conducted using digital image manipulation to create a 10-point PCR grading scale (10-100 in increments of 10). The scale was developed through Adobe Photoshop modification of high-resolution ocular photographs and validated by trained ophthalmic professionals. A total of 24 eyes from 12 patients were assessed using both the newly developed PCR scale and the established Schulze bulbar conjunctival redness (BCR) scale. Statistical analysis included Cronbach's alpha for internal consistency, intraclass correlation coefficients (ICC) for inter-rater reliability, paired t-tests for comparison between PCR and BCR scores, and Pearson's correlation coefficient to assess the relationship between the two measures.

Results: The PCR scale demonstrated excellent inter-rater reliability with an ICC of 0.773 (95% CI: 0.537-0.896) and strong internal consistency with a Cronbach's alpha of 0.880. Individual components showed robust reliability ranging from 0.842 to 0.891. Mean PCR scores were significantly higher than BCR scores by 15.90 units (p<0.0001), with a moderate correlation coefficient of 0.45 between the two measures. The combined PCR and BCR assessment achieved an ICC of 0.852 (95% CI: 0.780-0.902).

Conclusion: This study successfully established a reliable and clinically applicable PCR grading scale that addresses the unique anatomical and immunological characteristics of the palpebral conjunctiva. The moderate correlation between PCR and BCR measurements supports the necessity of independent assessment tools for different conjunctival regions. The PCR scale complements existing BCR assessments and provides enhanced diagnostic sensitivity for conditions affecting the eyelid margins and inner conjunctival surfaces. Further validation in larger, more diverse patient populations is recommended before widespread clinical implementation.

## Introduction

Ocular hyperemia is a key sign of inflammation and a common indicator of various eye conditions [1], but its clinical evaluation can be complex, especially when different regions of the conjunctiva are involved. The palpebral conjunctiva, located on the inner surface of the eyelids, has a distinct vascular supply compared to the bulbar conjunctiva, which covers the sclera.

The blood supply to the conjunctiva follows distinct anatomical pathways depending on its location. The palpebral portion, found on the eyelids' inner surface, receives its vascular supply through the marginal and peripheral arterial arcades - branches that stem from the ophthalmic artery [2]. Meanwhile, the bulbar conjunctiva overlying the white sclera is nourished by the anterior ciliary arteries, which, although originating from the same ophthalmic artery, take a different anatomical route [2]. These distinct vascular patterns can influence how different pathologies manifest eyelid conditions often present with palpebral conjunctival inflammation, whereas anterior segment disorders or systemic vascular conditions frequently affect the bulbar conjunctival vessels [2]. The difference in vascular supply between the bulbar and palpebral conjunctiva can lead to variation in the presentation of ocular inflammation, making it essential to distinguish palpebral conjunctival redness (PCR) from bulbar conjunctival redness (BCR) for nuanced diagnostics to guide appropriate treatment. Shumway et al. described that the palpebral conjunctiva, which lines the inner eyelid, contains a higher density of lymphatic vessels and lymphoid tissue compared to the bulbar conjunctiva, emphasizing its anatomical differences and its pivotal role in ocular immunity [3].

Furthermore, Knop and Knop (2005) described how bulbar and palpebral conjunctival lymphoid tissue show notable anatomical and functional variation. The palpebral conjunctiva, particularly in its tarsal and orbital zones, demonstrates a significantly higher density of lymphoid tissue compared to the bulbar region [4]. Other zones of the eye include the marginal, limbal, and fornical zones. This distribution includes both diffuse and organized lymphoid structures, with a notable abundance of plasma cells and high endothelial venules (HEVs) that mediate robust immune responses. This concentrated lymphatic presence suggests that the palpebral conjunctiva serves as a primary site for ocular surface immune defense. We hypothesize that increased density of HEVs and plasma cells in the palpebral conjunctiva means that evaluation of PCR offers a more sensitive approach to the detection of inflammatory or infectious conditions as compared with the use of BCR alone.

Understanding these anatomical and functional distinctions between palpebral and bulbar conjunctiva reinforces the necessity of a standardized PCR grading scale for clinical assessment. In 2007, Schulze et al. developed a grading scale for BCR that demonstrated high levels of repeatability [5]. An adaptation of this scale is shown in the Materials and Methods section. Despite the availability of grading scales for BCR5, a standardized, clinically validated scale for PCR has yet to be established. The present study aims to develop such a scale to address unique characteristics of palpebral redness, as opposed to solely bulbar redness, to improve diagnostic accuracy and sensitivity in both clinical practice and research.

## Materials and methods

**Figure 1:**
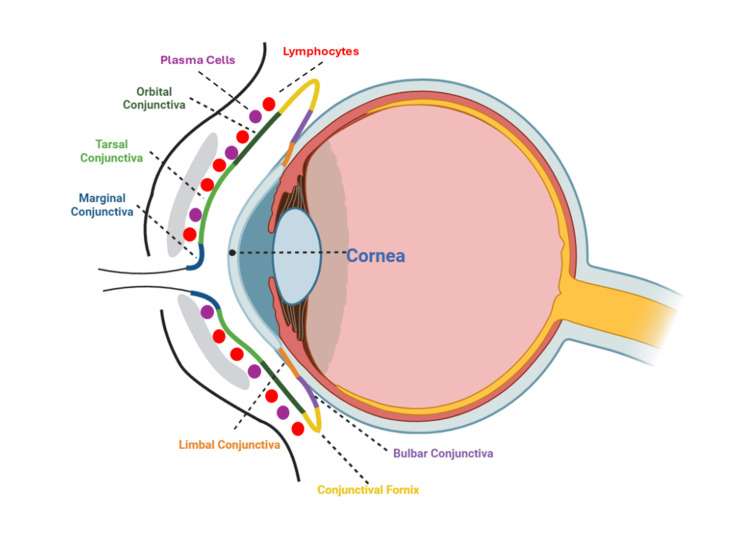
The image illustrates the different regions of the conjunctiva, divided into the palpebral conjunctiva, which consists of the marginal (dark blue), tarsal (bright green), orbital conjunctiva (dark green), the scleral conjunctiva, which consists of the bulbar conjunctiva (purple), limbal conjunctiva (orange), and the conjunctival fornices (yellow). The diagram also depicts the presence of plasma cells and lymphocytes in the conjunctival tissue as purple and red dots, demonstrating its immunological function. All components are color-coded for the reader's convenience. Original image created by the authors.

Figure 1 shows the other zones of the eye: the marginal, limbal, and fornical zones, as adapted from Knop and Knop (2005) [4].

**Figure 2:**
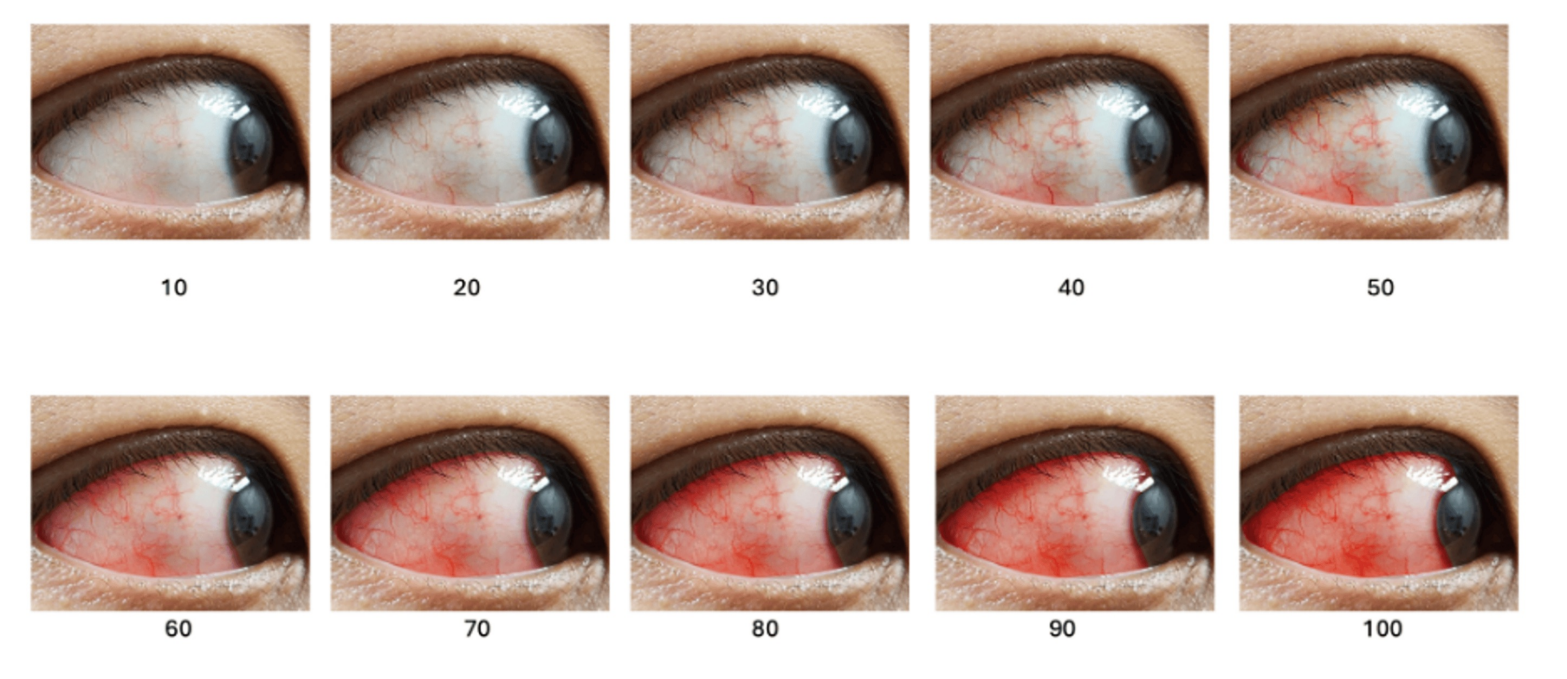
Standardized scale used to evaluate BCR, showing gradations from 10 to 100 in increments of 10. Adapted from Schulze's scale. Original image created by the authors. Source: [5].

This study focused on creating a PCR scale through digital image manipulation of controlled variation in redness levels. An initial high-resolution photograph of the eye was modified via Adobe Photoshop to generate 10 distinct gradations of PCR. These gradations ranged from 10 to 100 in increments of 10, progressively increasing the redness of the palpebral part of the eye. The created PCR scale was modeled after the standardized BCR scale found in the study by Schulze et al. [5], which was used to evaluate BCR, showing gradations from 10 to 100 in increments of 10. Redness intensification was achieved by selectively adjusting the hue and saturation of conjunctival blood vessels in the palpebral region using Adobe Photoshop. The red channel was enhanced incrementally while maintaining natural tissue tones and avoiding over-saturation artifacts. Each gradation represented an approximate 10% increase in vascular prominence compared to the baseline image (Figure 2).

Subsequently, the image scale was independently reviewed by three ophthalmic professionals with clinical experience in anterior segment examination to ensure accurate representation of the clinical appearance of PCR. Prior to formal assessment, raters reviewed both the newly developed PCR scale and Schulze's BCR scale to familiarize themselves with the grading methodology. Next, 24 eyes from 12 patients were assessed over multiple weeks opportunistically by trained raters using the newly developed PCR scale, while BCR was assessed using an established scale from Schulze et al. [5]. No specific inclusion or exclusion criteria were applied; patients were selected based on availability and willingness to participate, representing a convenience sample with varying degrees of conjunctival redness. Demographic data were not systematically collected for this pilot study.

All statistical analyses were performed using STATA software version 18 (StataCorp. 2023. Stata Statistical Software: Release 18. College Station, TX: StataCorp LLC). The sample size was determined based on feasibility for this pilot study; formal power analysis was not conducted, given the exploratory nature of scale development. Statistical analyses were performed with Cronbach's alpha to assess internal consistency and the intraclass correlation coefficient (ICC) to determine inter-rater reliability [6,7]. Cronbach's alpha determines the internal consistency and interrelatedness of items within a scale, with values above 0.70 indicating good reliability [8-10]. Similarly, the ICC assesses the consistency of assessments made by different observers for the same subject, with higher values indicating greater reliability [11,12]. This method of combining digital manipulation with clinical evaluation ensured that the scale was both theoretically sound and practically applicable.

Additionally, a paired t-test was conducted to compare mean BCR and PCR scores. Pearson's correlation coefficient was then calculated to assess the relationship between PCR and BCR. These statistical methods were done to support the distinctiveness and reliability of the newly developed PCR scale.

## Results

**Figure 3:**
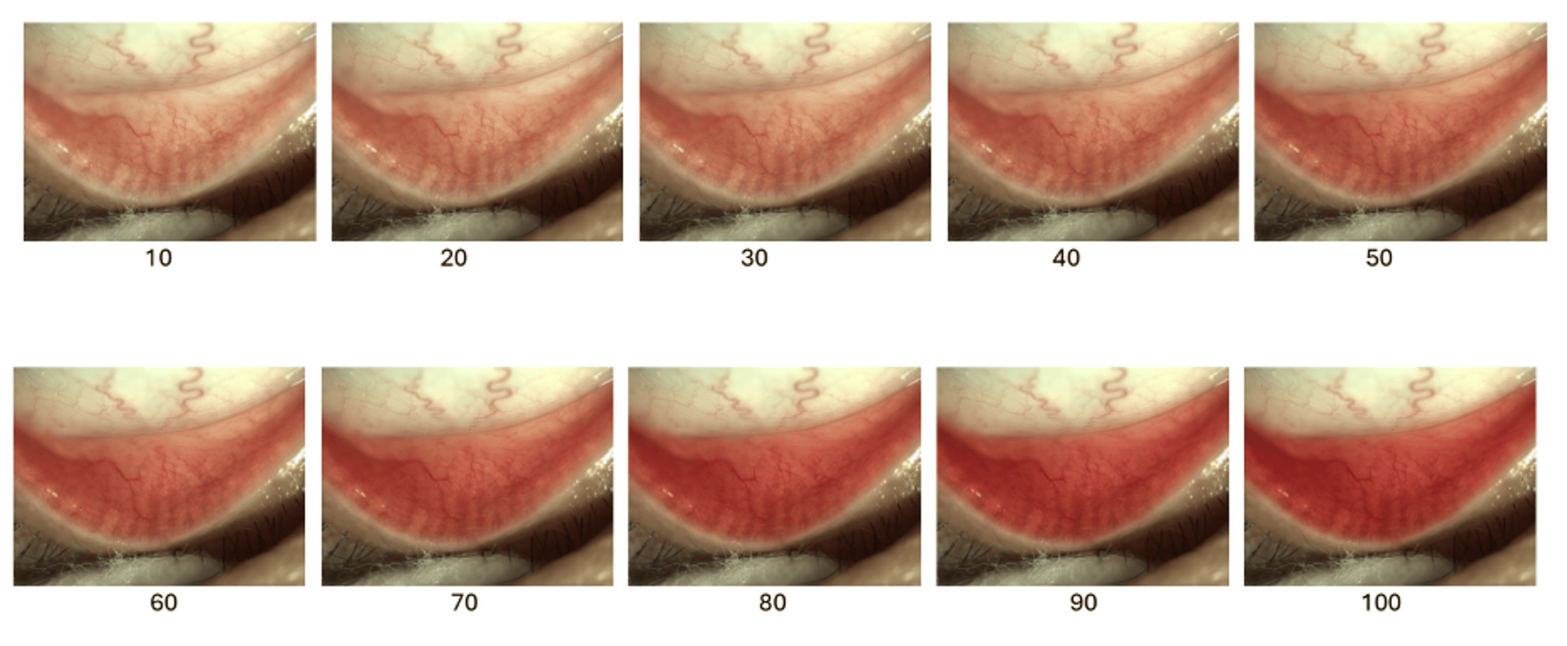
Standardized scale used to evaluate palpebral conjunctival redness (PCR), showing gradations from 10 to 100 in increments of 10. Original image created by the authors.

The PCR scale represents 10 gradations of redness intensity, providing a spectrum of redness from minimal (10) to severe (100) (Figure 3). Inter-rater reliability was robust across PCR and BCR assessments, with ICC values for PCR at 0.773 (95% CI: 0.537-0.896), BCR at 0.802 (95% CI: 0.624-0.893), and a combined score of 0.852 (95% CI: 0.780-0.902). BCR was assessed using Schulze's panel, further demonstrating the robust validity of Schulze's BCR grading method [5]. Tables 1, 2 demonstrate the inter-rater reliability and internal consistency.

**Table 1:** Inter-rater reliability assessment using intraclass correlation coefficients (ICC) for palpebral conjunctival redness (PCR), bulbar conjunctival redness (BCR), and combined assessments. Values demonstrate excellent agreement between evaluators, with 95% confidence intervals indicating robust statistical reliability across all conjunctival regions evaluated.

Area	ICC Value	95% Confidence Interval
PCR	0.773	0.537–0.896
BCR	0.802	0.624–0.893
Combined	0.852	0.780–0.902

**Table 2:** Internal consistency analysis using Cronbach's alpha coefficients for individual components of the conjunctival redness assessment scale. Values represent reliability measures for right eye (OD) and left eye (OS) palpebral conjunctival redness (PCR), as well as nasal and temporal bulbar conjunctival redness (BCR) assessments, demonstrating strong internal consistency across all measured parameters.

Area	Cronbach Alpha
PCR OD	0.872
PCR OS	0.891
BCR OD Nasal	0.847
BCR OD Temporal	0.851
BCR OS Nasal	0.845
BCR OS Temporal	0.842
Total	0.880

## Discussion

Our findings highlight the importance of a standardized PCR scale in addressing the unique clinical presentation of PCR. The excellent inter-rater reliability, as indicated by an ICC of 0.852, supports the scale's robustness and suggests that each item (e.g., PCR OD, BCR OS nasal) is appropriately aligned with the overall construct of conjunctival redness, providing a rationale for the scale's applicability across different clinical settings. The high correlation among raters indicates that the digital manipulation method created a reliable spectrum that clinicians found accurate in representing real-world PCR levels.

BCR was evaluated using Schulze's panel [5]. Each component of the scale, including PCR for the right (OD) and left (OS) eyes and BCR for the nasal and temporal regions in both eyes, displayed strong internal consistency. Individual Cronbach's alpha values for each item ranged from 0.8423 to 0.8911, showing that each region contributes robustly to the scale's overall reliability [11,12]. This high internal consistency suggests that the scale provides a dependable measure for assessing conjunctival redness across the palpebral and bulbar regions.

Importantly, the PCR scale complements existing BCR grading methods, enabling a more comprehensive assessment of conjunctival redness. Additionally, these results emphasize the need for specialized tools when evaluating different regions of ocular redness, as distinct vascular patterns can affect how redness appears and is perceived. Our own clinical observations demonstrate that PCR often may not correlate with BCR. This incongruity between PCR and BCR presentations suggests that these manifestations may represent distinct pathophysiological processes, further emphasizing the need for independent grading scales for each type of conjunctival redness.

In anterior blepharitis, the conjunctival segments are affected asymmetrically, with the palpebral conjunctiva bearing the primary impact. Positioned to receive direct exposure, the palpebral conjunctiva is especially vulnerable to anterior blepharitis, which manifests as redness, swelling at the eyelash base, lid margin crusting, and symptoms of burning and irritation [13]. The anatomical susceptibility of the palpebral conjunctiva is due to its vascular supply from the eyelid’s marginal and peripheral arcades. In contrast, bulbar redness may not reveal itself in lid margin disease until there is significant tear film dysfunction as a sequela of lid margin disease [14]. Supplied by the anterior ciliary arteries, the bulbar conjunctiva is more distal to the primary inflammatory site in lid margin disease. Clinically, palpebral hyperemia has often been observed to be incongruous with bulbar hyperemia, further supporting the anatomical difference in disease presentation of palpebral and bulbar conjunctiva.

Beyond anterior blepharitis, the PCR scale has significant utility in other high-yield clinical scenarios where detection of palpebral inflammation is critical for diagnosis and management. In allergic conjunctivitis and vernal keratoconjunctivitis, PCR measurement can help differentiate inflammatory conditions from infectious etiologies and guide treatment decisions. The palpebral conjunctiva exhibits prominent papillary reactions in vernal keratoconjunctivitis, particularly on the superior tarsal conjunctiva, making PCR assessment valuable for disease monitoring and treatment response evaluation [15]. In trachoma, caused by *Chlamydia trachomatis*, PCR grading could potentially provide an objective measure for disease surveillance and monitoring treatment efficacy in endemic regions, where the characteristic follicular response predominantly affects the tarsal conjunctiva [16]. Additionally, viral conjunctivitis, particularly adenoviral keratoconjunctivitis, often presents with marked palpebral injection that may precede or exceed bulbar involvement, making PCR assessment a sensitive early indicator of disease activity. The higher density of lymphoid tissue and plasma cells in the palpebral conjunctiva, as previously discussed, supports its role as a more sensitive indicator of immune-mediated responses across these diverse ocular surface conditions. This broad applicability reinforces the clinical value of standardized PCR measurement beyond eyelid-specific pathology.

The results of the paired t-test indicate that the mean BCR score was lower than the PCR score by 15.90 units (p<0.0001). This reinforces that PCR generally presents with greater redness than BCR. While this difference may reflect genuine anatomical and vascular variations between the palpebral and bulbar conjunctiva, we acknowledge that scale calibration factors may also contribute to this finding. The PCR scale was developed through digital manipulation of palpebral conjunctival images, while BCR was assessed using Schulze's independently developed scale. These scales, though both ranging from 10 to 100, were created from different baseline images and may have inherent differences in how redness intensity is represented across the spectrum. Future studies should investigate whether this numeric disparity reflects true biological differences in redness presentation, methodological factors related to scale development, or a combination of both. Calibration studies comparing the two scales against objective measures of vascular density or blood flow could help disentangle these factors. The moderate correlation demonstrates that the two scales only moderately align, reinforcing the necessity of having an independent PCR grading scale rather than relying solely on previous BCR scales or assessments. Moreover, a Pearson correlation coefficient of 0.45 was found, which indicates a moderate correlation between BCR and PCR. This finding highlights that while there is some association between the two measures, BCR does not always reliably predict PCR values.

Incorporating insights from Ogawa et al., we emphasize the necessity of using dual grading scales, particularly in chronic ocular graft-versus-host disease (cGVHD) models [17]. The article underscores that the nuanced clinical presentations of cGVHD demand independent evaluation methods for different conjunctival regions due to varying vascular supplies and disease presentations.

While our study provides a standardized scale for measuring PCR and BCR, we have not yet established definitive frameworks for what constitutes normal ranges in either measurement. Preliminary observations suggest that normal BCR values might fall within 10-30, while normal PCR values may range from 10 to 40, with values above these ranges potentially indicating abnormalities or pathological conditions. However, these proposed ranges require validation through future research to establish clinical guidelines for distinguishing between normal and abnormal presentations of conjunctival redness. Nevertheless, the scales offer significant value as tools for tracking changes over time and assessing treatment responses. Even if their utility in initial diagnosis is currently limited, they provide an objective framework for monitoring disease progression and evaluating the effectiveness of therapeutic interventions.

Utilizing both the PCR and BCR grading scales provides a more comprehensive means of assessment that can capture distinct characteristics of each conjunctival region. Future studies should explore the practical applications of this scale in more diverse clinical settings, with the aim to further refine and validate the scale for broader use.

Limitations

Given the limited sample size of 24 eyes from 12 patients, this study faces several important constraints. The small cohort does not adequately represent the diverse spectrum of PCR presentations encountered in clinical practice, potentially limiting the scale's generalizability across different patient populations, age groups, and ethnicities. The lack of systematic demographic data collection (age, sex, ethnicity) limits our ability to assess whether the scale performs consistently across diverse populations. Future validation studies should include a comprehensive demographic characterization to ensure broad applicability. Additionally, the methodology of developing the scale using digital manipulation of a single initial photograph may not fully capture the natural variations in PCR manifestation. The convenience sampling method and lack of specific inclusion/exclusion criteria limit the generalizability of our findings. The brief training protocol for raters, while sufficient to demonstrate scale usability, may not reflect the reliability achievable with more extensive standardized training. Additionally, the heterogeneous levels of clinical experience among raters may have introduced variability, though the strong ICC values suggest this did not substantially impact reliability.

Furthermore, this study focuses solely on the inferior palpebral conjunctiva, while the superior palpebral conjunctiva presents different characteristics and was not evaluated. This limitation reduces the scales’ generalizability to the upper lid. However, clinical examination of the lower lid is usually more expeditious, as upper-lid tarsal eversion is relatively rare in routine clinical settings. While trained raters were used for validation, the study's pilot nature means that long-term reliability and clinical utility remain to be established. These limitations underscore the need for larger-scale validation studies and continued refinement of the scale before it can be recommended for widespread clinical implementation.

## Conclusions

This pilot study successfully developed and validated a standardized PCR scale that offers a potentially reliable method for assessing PCR. By addressing the unique vascular characteristics and clinical presentation of PCR, this scale may enhance the accuracy and consistency of ocular redness evaluations in clinical settings, pending validation in larger, more diverse populations. While the PCR scale complements existing BCR assessments, further research with rigorous methodology, larger sample sizes, and diverse patient demographics is needed to establish clinical utility and integrate both scales into routine practice. The strong inter-rater reliability and internal consistency demonstrated in this preliminary work support continued development and validation of this tool. Future studies should also investigate whether the observed differences between PCR and BCR reflect true anatomical distinctions or are influenced by scale calibration factors and should establish normative ranges for clinical interpretation.
